# Association between “Life's Essential 8” cardiovascular health and apparent treatment-resistant hypertension among US adults from the NHANES, 2005 to 2018

**DOI:** 10.3389/fcvm.2024.1453563

**Published:** 2024-12-13

**Authors:** Zhong-jiao Xu, Ru-ming Shen, Wu-ming Hu, Jia-yi Shen, Xiao-yan Wu, Ling-chun Lv

**Affiliations:** Department of Cardiology, The Fifth Affiliated Hospital of Wenzhou Medical University, Lishui Central Hospital, Zhejiang, China

**Keywords:** cardiovascular health score, healthy lifestyle, life's essential 8, hypertension, apparent treatment-resistant hypertension (aTRH)

## Abstract

**Background:**

The association between healthy lifestyle and American Heart Association (AHA) Life's Essential 8 (LE8) score and apparent treatment-resistant hypertension(aTRH)remains uncertain. We aimed to explore the association between healthy lifestyle and higher LE8 score and apparent treatment-resistant hypertension in the general population.

**Methods:**

Using NHANES data from 2005 to 2018, we included and analyzed information on 7,474 participants eligible for this study. The association between LE8 and aTRH was explored using logistic regression models, and the association between LE8 and antihypertension drugs uncontrolled hypertension was further explored using logistic regression models.

**Results:**

Participants with higher LE8 scores tended to be non-Hispanic white and married or living with a partner; have low income and higher education; and be without Chronic kidney disease (CKD)(all *p*-values <0.001). Compared to subjects with low CVH, participants with moderate and high CVH exhibited lower risks of 47% and 76%, respectively. After adjusting for covariates, there was no evidence of a nonlinear association between LE8 and aTRH (*p* for nonlinearity = 0.456). Physical activity (PA), body mass index (BMI), and blood glucose were associated with aTRH (all *p*-values < 0.05), while diet, nicotine exposure, sleep, and blood lipids were not significantly associated with aTRH. Compared to the low LE8 group, the ORs for the high LE8 group were 0.46 (95% CI, 0.28 to 0.76) and 0.07 (95% CI, 0.02 to 0.20) for uncontrolled hypertension with 1–2 and 3–4 antihypertensive drugs, respectively. In the sensitivity analysis, subgroup analyses were performed on all covariates, and the results remained stable.

**Conclusion:**

In our study, we found a significant association between higher LE8 scores and a lower risk of aTRH. Our findings suggest that implementing various healthy lifestyle practices and managing known cardiovascular risk factors could be a feasible comprehensive preventive approach to aTRH.

## Introduction

Hypertension is the most prevalent cardiovascular disease worldwide. According to the data presented in the first-ever “Global Report on Hypertension” released by the World Health Organization (1), the number of global hypertension patients has risen from 650 million in 1990 to 1.3 billion in 2019 over the past 30 years. Alarmingly, nearly half of these individuals are unaware of their condition, with 82% of them residing in low-income and middle-income countries (1, 2). While diagnosing hypertension is relatively straightforward and manageable with low-cost medication interventions, research indicates that approximately 580 million individuals with hypertension remain undiagnosed (41% female patients, 51% male patients), and over half of those diagnosed with hypertension (53% female and 62% male), totaling 720 million people, do not receive necessary interventions (2). Elevated blood pressure is closely associated with the occurrence and progression of diseases such as stroke, coronary heart disease, heart failure risk, atrial fibrillation, and chronic kidney disease (3, 4). Moreover, it is estimated that annually, high systolic blood pressure contributes to over 10 million global fatalities, surpassing deaths caused by other health risks (1). In China, cardiovascular diseases, predominantly stroke and ischemic heart disease, rank as the leading cause of mortality among the Chinese population. Among patients with cardiovascular disease in China, the highest number of cases is attributed to hypertension.

Apparent treatment-resistant hypertension (aTRH) represents a severe form of hypertension, with a higher incidence of target organ damage, such as CKD (5, 6) and left ventricular hypertrophy (7). aTRH is also a risk factor for both CKD and cardiovascular diseases (8), including myocardial infarction and stroke, particularly heart failure (9). In contrast, patients with resistant hypertension exhibit higher risks of cardiovascular events and all-cause mortality rates (10, 11), with a nearly twofold increased relative risk of cardiovascular events (12). Owing to variations in data from different populations, determining the precise prevalence of resistant hypertension remains challenging. However, recent publications suggest a prevalence range of 6%–18% among hypertensive patients (6). Despite the existence of safe and effective treatment methods, including lifestyle interventions, antihypertensive medication therapies, and more recently, catheter-based renal artery denervation (13), the awareness of the condition and the achievement of blood pressure control rates recommended by guidelines remain suboptimal (2).

In 2010, the American Heart Association (AHA) introduced a novel cardiovascular health framework, aiming to facilitate a paradigm shift from a sole focus on disease treatment to encompassing the promotion and protection of health throughout both the population and individual lifespans. This initiative led to the establishment of “Life's Simple 7(LS7),” consisting of seven ideal targets encompassing health behaviors and factors, namely, healthy diet, physical activity(PA), normal body mass index (BMI), nonsmoking, normal blood pressure(BP), normal fasting blood glucose, and normal total cholesterol, all intended to promote ideal CVH (14). Despite the favorable effects observed in promoting CVH through the implementation of interventions targeting these factors, the prevalence of ideal CVH remains exceedingly low in the American population (<1%) (15). According to relevant research, limitations persist within Life's Simple 7, including a limited range of health behaviors (e.g., sleep health was not included), a rudimentary additive scoring system, and low sensitivity to interindividual and intraindividual variations (16). To overcome the limitations of LS7 and consider an expanded age range across the entire lifespan, as well as the fundamental background of social determinants of physical and psychological health, the AHA recently recommended “Life's Essential 8” (LE8) to maintain optimal CVH. LE8 incorporates sleep health as an additional component and updates and refines the other seven components of LS7, particularly altering the component scoring algorithm within LE8. Each indicator now has a new scoring algorithm, allowing for the generation of a new composite cardiovascular health score. For ease of reference in clinical or research settings, the eight indicators have been divided into two domains: health behaviors (diet, physical activity, nicotine exposure, sleep) and health factors (body mass index, blood lipids, blood glucose, blood pressure).

aTRH is a multifactorial disease, a more severe and complex form of hypertension associated with higher cardiovascular event risks. prior research that predominantly centered on general hypertension risk, and it remains unclear whether the ideal levels of the components within LE8 are associated with improved blood pressure control and a lower risk of resistant hypertension. Establishing the relationship between cardiovascular health and the risk of resistant hypertension may aid in reducing the risk of aTRH in adult hypertensive patients without the need for additional antihypertensive medications. Our aim was to explore the association between the recently published cardiovascular health metric LE8 and aTRH.

## Methods

### Population

**Figure 1:**
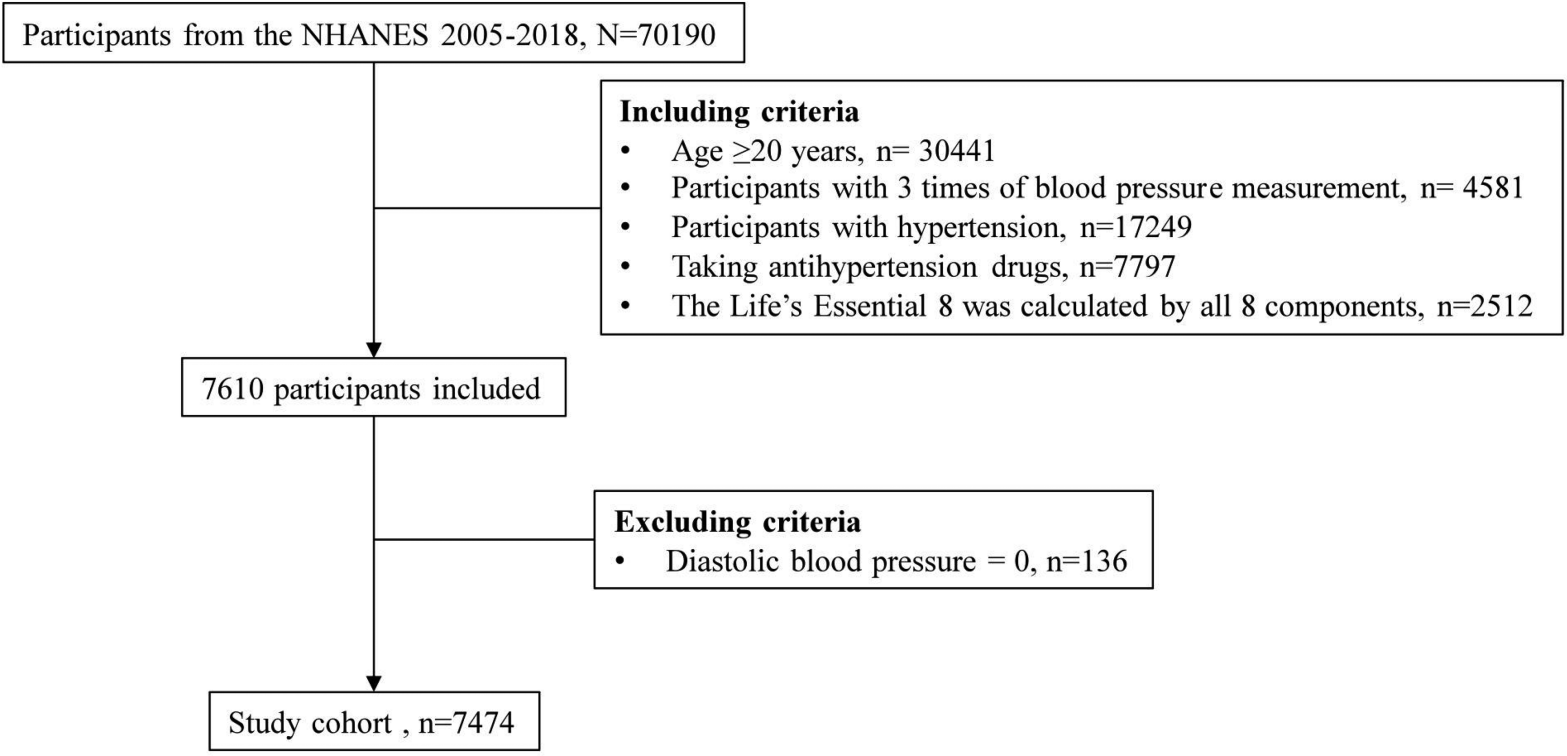
Flowchart of participants selection.

A total of 70,190 participants from the NHANES 2005–2018 waves were recruited due to the inclusion of sleep health information, which was first collected in the 2005–2006 NHANES (17). The inclusion criteria were as follows: age 20 years or older, with 3 blood pressure measurements, hypertension, antihypertensive drug use, and all 8 components of LE8. NHANES allowed diastolic blood pressure (BP) to be 0, so participants with diastolic blood pressure of 0 were excluded (Figure 1). After applying the aforementioned inclusion and exclusion criteria, no pregnant women (i.e., self-reported their pregnancy at the time of the examination or had a positive result on the urine test) were included in the study population. The NHANES program was approved by the National Center for Health Statistics (NCHS) Ethics Review Board, and all participants provided written informed consent. Since NHANES data are deidentified and anonymous during analysis, no additional ethical approval or informed consent is needed for secondary analyses.

### Blood pressure, antihypertensive drugs, hypertension, and resistant hypertension

BP measurements were conducted in individuals who did not exhibit any of the following conditions: rashes, gauze dressings, casts, edema, paralysis, tubes, open sores or wounds, withered arms, arterial-ventricular shunts, or had undergone a radical mastectomy. Prior to the commencement of BP measurements, an assessment of the upper arm circumference was performed with the objective of determining the appropriate cuff size to be employed. Subsequently, participants were instructed to remain seated in a quiet and relaxed state for a duration of 5 min. Then, three consecutive BP readings were recorded. In the event of an interruption or incomplete recording during any BP measurement, a fourth attempt was permitted (18).

During the household interview, skilled interviewers inquired whether participants had consumed prescription medications over the past 30 days that necessitated a prescription. Respondents answering in the affirmative were requested to present the interviewer with the containers of the medications they had used. In cases where no container was available, participants were prompted to verbally communicate the names of the medications (19). The categories of antihypertensive medications are outlined in Supplementary Table S1 (20).

Hypertension was diagnosed if participants had previously received a diagnosis of hypertension, if their mean blood pressure measurements exceeded or equaled 130/80 mmHg, or if they were currently on antihypertensive agents. Incident aTRH was defined as a systolic blood pressure (SBP) reading of 130 mmHg or higher, a diastolic blood pressure (DBP) reading of 80 mmHg or higher while taking a minimum of three different classes of antihypertensive medication, or taking four or more different classes of antihypertensive medication with an SBP lower than 130 mmHg and a DBP lower than 80 mmHg (21, 22).

### Life's essential 8

The LE8 score is calculated using 4 health behaviors (diet, physical activity, smoking, and sleep) and 4 health factors [body mass index, non–high density lipoprotein (HDL) cholesterol, blood glucose, and BP].

Dietary adherence to the Healthy Eating Index 2015 was assessed through two interviewer-administered 24-h dietary recalls (23), utilizing the Food Patterns Equivalents Database (24) [formerly MyPyramid Equivalents Database (25)] to determine servings of dietary components. Self-reported PA was assessed by inquiring about the frequency and duration of recreational physical activities over the past 30 days, categorized as either vigorous (heavy sweating or large increases in breathing or heart rate) or moderate (light sweating or small increase in breathing or heart rate). Smoking status was determined through questionnaires on cigarette use and recent usage of e-cigarettes and other tobacco products. Exposure to secondhand smoke for all household members was evaluated using the family household questionnaire. Sleep health was evaluated through interviewer-administered questions about sleep habits and disorders.

BMI was calculated using standardized height and weight measurements. Blood samples were collected and sent to central laboratories for analysis of blood lipids, plasma glucose, and hemoglobin A1c. BP was manually measured after 5 min of seated rest in a quiet room, with the average of 3 measurements recorded. Medication usage was assessed during the home interview. More detailed calculations are presented in Supplementary Table S2.

Each of the 8 components was scored on a scale from 0 to 100. The individual LE8 score was then calculated by summing these component scores and dividing the total by 8, yielding a score ranging from 0 to 100 (16). Following American Heart Association (AHA) recommendations, overall LE8 scores were categorized into low LE8 (0–49 points), moderate LE8 (50–79 points), and high LE8 (80–100 points) (16).

### Covariates

Demographic information, including age, sex, ethnicity, income-to-poverty ratio (PIR), marital status, and education level, was obtained from the demographics file. Ethnicity was classified as Mexican American, Non-Hispanic Black, Non-Hispanic White, or other (26). The PIR is an index of income levels, where ≤1.3 indicates low income, 1.3–3.5 indicates median income, and >3.5 indicates high income (27). Marital status was categorized as married or living with a partner and not married or living with a partner (26). Education levels were classified as less than high school and equal to or above high school (26). Health care visit frequency in the past year was included as a covariate since ≥4 visits annually has been identified as a potential risk factor for aTRH (28). CKD were diagnosed as Chronic Kidney Disease Epidemiology Collaboration (CKD-EPI) equation-calculated estimated glomerular filtration rate (eGFR) < 60 ml/min/1.73 m^2^ or previously diagnosed weak/failing kidneys (29).

### Statistics

A considerable proportion of missing data was observed for the PIR variable (8.13%, *n* = 608) (Supplementary Figure S1). To retain the maximum sample size, missing values were imputed utilizing the random forest method through the mice package in R software. Continuous variables were summarized using the median (interquartile range, IQR) based on the distribution, while categorical variables were presented as frequencies (percentages). Between-group comparisons for continuous variables were conducted using the Kruskal‒Wallis rank sum test, and Pearson's chi-square test was utilized for categorical variables. A *p*-value < 0.05 was considered indicative of statistical significance.

Logistic regression models were utilized to investigate the association between LE8 and aTRH. Model 1 was unadjusted, while Models 2 and 3 included the following covariates: age, sex, ethnicity, marital status, income level, health care utilization frequency, educational attainment, and data collection waves (Model 2); and Model 2 covariates plus chronic kidney disease status (Model 3). To explore potential nonlinear relationships between LE8 and aTRH, a restricted cubic spline (RCS) logistic regression model with 5 knots was fitted using the 5th, 27.5th, 50th, 72.5th, and 95th percentiles of LE8 while adjusting for all covariates (Model 3). The 100th percentile of LE8 (97.50) served as the reference. Subgroup analyses were conducted across all covariates to further elucidate the association between LE8 and aTRH in different models.

In supplementary analyses, logistic models were further used to explore the associations of LE8 with the use of 1–2 antihypertensive drugs with uncontrolled hypertension in all included participants and the use of 3–4 antihypertensive drugs with uncontrolled hypertension in participants with aTRH. Additionally, we further explored the correlations between each component of LE8 and aTRH.

Multiple sensitivity analyses were conducted. First, the blood pressure threshold defining aTRH was set to 140/90 mmHg. Second, complete-case analyses were performed to avoid potential bias from data imputation. Third, the LE8 score was standardized, and the odds ratio (OR) with a 95% confidence interval per standard deviation (SD, 13.18) increase in the continuous LE8 score was calculated. Finally, the association between LE8 without the blood pressure component and aTRH was investigated.

## Results

Table 1 presents the baseline characteristics of the 7,474 participants included in the study. Of the participants, 52.65% were female and 48.11% were non-Hispanic white, with a median age of 64.00 years (IQR, 19.00). Participants with higher LE8 scores tended to be non-Hispanic white, married or living with a partner, have low income, attain higher education, and be without CKD (all *p*-values <0.001). Interestingly, participants with higher LE8 scores were older in our study (*p* < 0.001).

**Table 1:** Baseline characteristics based on life's essential 8 levels.

Life's Essential 8					
Characteristic	Overall, *N* = 7,474^a^	Low, *N* = 1,849^a^	Median, *N* = 5,262^a^	High, *N* = 363^a^	*p*-value^b^
Age, y	64.00 (19.00)	62.00 (18.00)	65.00 (18.00)	66.00 (16.00)	<0.001
Gender					0.013
Female	3,935 (52.65%)	1,028 (55.60%)	2,717 (51.63%)	190 (52.34%)	
Male	3,539 (47.35%)	821 (44.40%)	2,545 (48.37%)	173 (47.66%)	
Ethnicity					<0.001
Other	1,128 (15.09%)	250 (13.52%)	790 (15.01%)	88 (24.24%)	
Non-Hispanic White	3,596 (48.11%)	762 (41.21%)	2,635 (50.08%)	199 (54.82%)	
Non-Hispanic Black	1,981 (26.51%)	629 (34.02%)	1,294 (24.59%)	58 (15.98%)	
Mexican American	769 (10.29%)	208 (11.25%)	543 (10.32%)	18 (4.96%)	
Marital Status					<0.001
Not Married nor Living With a Partner	2,996 (40.09%)	865 (46.78%)	2,030 (38.58%)	101 (27.82%)	
Married or Living With a Partner	4,478 (59.91%)	984 (53.22%)	3,232 (61.42%)	262 (72.18%)	
Income Levels					<0.001
Low	2,265 (30.31%)	309 (16.71%)	1,755 (33.35%)	201 (55.37%)	
Median	3,034 (40.59%)	775 (41.91%)	2,143 (40.73%)	116 (31.96%)	
High	2,175 (29.10%)	765 (41.37%)	1,364 (25.92%)	46 (12.67%)	
Education Levels					<0.001
Less than High School	1,911 (25.57%)	644 (34.83%)	1,223 (23.24%)	44 (12.12%)	
High School Graduate or Higher	5,563 (74.43%)	1,205 (65.17%)	4,039 (76.76%)	319 (87.88%)	
Healthcare Visit Times Last Year					<0.001
<4	2,835 (37.93%)	547 (29.58%)	2,112 (40.14%)	176 (48.48%)	
≥4	4,639 (62.07%)	1,302 (70.42%)	3,150 (59.86%)	187 (51.52%)	
Chronic Kidney Diseases					<0.001
No	5,668 (75.84%)	1,324 (71.61%)	4,034 (76.66%)	310 (85.40%)	
Yes	1,806 (24.16%)	525 (28.39%)	1,228 (23.34%)	53 (14.60%)	
Resistant Hypertension					<0.001
No	6,435 (86.10%)	1,461 (79.02%)	4,630 (87.99%)	344 (94.77%)	
Yes	1,039 (13.90%)	388 (20.98%)	632 (12.01%)	19 (5.23%)	
Uncontrolled Hypertension in 1∼2 Antihypertensive Drugs					<0.001
No	2,274 (30.43%)	344 (18.60%)	1,725 (32.78%)	205 (56.47%)	
Yes	5,200 (69.57%)	1,505 (81.40%)	3,537 (67.22%)	158 (43.53%)	
Uncontrolled Hypertension in 3∼4 Antihypertensive Drugs					<0.001
No	6,578 (88.01%)	1,499 (81.07%)	4,725 (89.79%)	354 (97.52%)	
Yes	896 (11.99%)	350 (18.93%)	537 (10.21%)	9 (2.48%)	

^a^ median (interquartile range, IQR); *n* (%).

^b^ Kruskal-Wallis rank sum test; Pearson's Chi-squared test.

**Figure 2:**
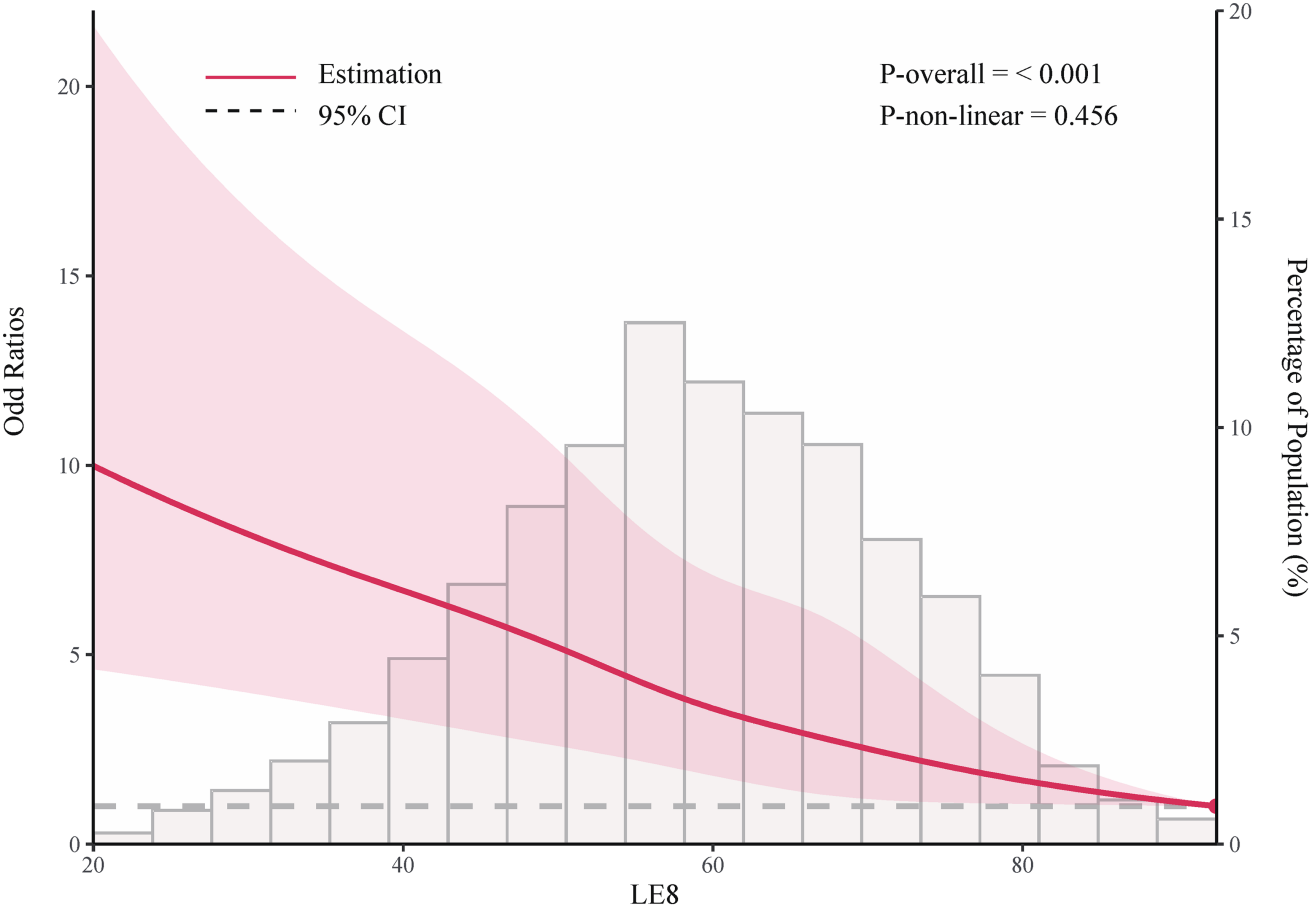
Adjusted odd ratio for the association between life's essential 8 and resistant hypertension. Data were fitted using logistic models with restricted cubic spline (RCS) with 5 knots (the 5.0th, 27.5th, 50th, 72.5th, and 95.0th percentiles) for life's essential 8 socre, adjusted for potential covariates. Reference is the 100th percentile of life's essential 8 socre (97.50).

As shown in Table 2, in the fully adjusted model (Model 3), compared to the low LE8 group, the ORs and 95% CIs for the median LE8 and high LE8 groups were 0.53 (95% CI, 0.46 to 0.62) and 0.24 (95% CI, 0.14 to 0.38), respectively. Figure 2 demonstrates no evidence of a nonlinear association between LE8 and aTRH (*p* for nonlinearity = 0.456). Supplementary Figure S2 delineates the outcomes of subgroup analyses, revealing a consistent association between LE8 and aTRH across diverse subpopulations. In comparison to individuals with a low LE8 score, those with a high LE8 score exhibited OR of 0.16 [95%CI, 0.06–0.43], 0.3 [95% CI, 0.17–0.52], 0.24 [95% CI, 0.12–0.49], 0.24 [95% CI, 0.12–0.47], 0.25 [95% CI, 0.13–0.46], 0.28 [95% CI, 0.16–0.46], and 0.09 [95% CI, 0.02–0.4] for participants aged <65 years, ≥65 years, female, male, non-Hispanic white, without CKD, and with CKD, respectively. No significant interactions were detected between LE8 and the covariates (all *p*-values for interaction > 0.05).

**Table 2:** Association between life's essential 8 and resistant hypertension in different models.

Life's essential 8	Model 1^a^	*P*-values	Model 2^a^	*P*-values	Model 3^a^	*P*-values
Low	Ref		Ref		Ref	
Median	0.51 [0.45,0.59]	<0.001	0.51 [0.44,0.6]	<0.001	0.53 [0.46,0.62]	<0.001
High	0.21 [0.13,0.33]	<0.001	0.22 [0.13,0.35]	<0.001	0.24 [0.14,0.38]	<0.001
*P*-values for Trend	<0.001		<0.001		<0.001	

^a^ odd ratios (OR) and 95% confidence intervals (CI) were calculated by logistic regressions. Model 1, unadjusted for covariates; Model 2, adjusted for age, gender, ethnicity, marital status, income level, healthcare visit times last years, educational level and data cycles; Model 3, Model 2 +chronic kidney diseases.

Table 3 presents the correlations between individual LE8 metrics and aTRH. PA, BMI, and blood glucose were associated with aTRH (all *p*-values < 0.05), while diet, nicotine exposure, sleep, and blood lipids were not significantly associated with aTRH. Compared to individuals with low levels of each metric, individuals with high levels had odds ratios (ORs) of 0.93 [95% CI, 0.79–1.10] for diet, 0.82 [95% CI, 0.71–0.94] for PA, 1.07 [95% CI, 0.88–1.31] for nicotine exposure, 0.94 [95% CI, 0.79–1.11] for sleep health, 0.51 [95% CI, 0.41–0.62] for BMI, 1.07 [95% CI, 0.93–1.25] for blood lipids, and 0.49 [95% CI, 0.41–0.57] for blood glucose.

**Table 3:** Association between life's essential 8 metrics and resistant hypertension.

Life's essential 8 metrics	Low	Median	High	*P*-values for Trend
Diet	Ref	0.91 [0.77,1.08]	0.93 [0.79,1.1]	0.369
*P*-value		0.296	0.419	
Physical activity (PA)	Ref	0.61 [0.41,0.89]	0.82 [0.71,0.94]	0.006
*P*-value		0.011	0.006	
Nicotine exposure	Ref	1.22 [0.99,1.5]	1.07 [0.88,1.31]	0.973
*P*-value		0.059	0.502	
Sleep health	Ref	1.02 [0.83,1.25]	0.94 [0.79,1.11]	0.381
*P*-value		0.882	0.462	
Body mass index	Ref	0.67 [0.58,0.79]	0.51 [0.41,0.62]	<0.001
*P*-value		<0.001	<0.001	
Blood lipids	Ref	0.84 [0.67,1.04]	1.07 [0.93,1.25]	0.292
*P*-value		0.105	0.345	
Blood glucose	Ref	0.64 [0.54,0.75]	0.49 [0.41,0.57]	<0.001
*P*-value		<0.001	<0.001	
Blood pressure (BP)	Ref	0.22 [0.17,0.28]	0.24 [0.19,0.3]	<0.001
*P*-value		<0.001	<0.001	

The results for the associations of LE8 with uncontrolled hypertension and the use of 1–2 or 3–4 antihypertensive drugs are presented in Supplementary Table S3. Compared to the low LE8 group, the ORs for the high LE8 group were 0.46 (95% CI, 0.28–0.76) and 0.07 (95% CI, 0.02–0.20) for uncontrolled hypertension with the use of 1–2 or 3–4 antihypertensive drugs, respectively.

In the model adjusted for all potential covariates, the odds ratios (ORs) for the population with high LE8, compared to those with low LE8, were 0.28 [95% CI, 0.17–0.47] and 0.5 [95% CI, 0.36–0.67] when using a blood pressure cutoff of 140/90 mmHg and when no blood pressure data were included in the calculation of LE8, respectively. In the complete-case analysis, the OR was 0.24 [95% CI, 0.14–0.39]. When LE8 was considered a continuous variable, the OR for every 1 SD change in LE8 was 0.64 [95% CI, 0.59–0.7].

## Discussion

In this large cross-sectional study, we evaluated the association between the level of CVH as defined by LE8 scores and the presence of aTRH. We observed a significantly lower risk of aTRH associated with higher levels of CVH. Compared to subjects with low CVH, participants with moderate and high CVH exhibited lower risks of 47% and 76%, respectively. After adjusting for covariates, there was no evidence to suggest a nonlinear association between LE8 and aTRH (nonlinear *p* = 0.456).

Our findings are consistent with the current research understanding. A community-based prospective Jackson Heart Study (JHS) (30), including 1,878 individuals without hypertension or cardiovascular disease at baseline, suggested a lower risk of hypertension among individuals with higher LS7 scores, specifically in relation to those with normal blood pressure and prehypertension. Better cardiovascular health, as indicated by improved health behaviors and factors, was associated with a decreased risk of hypertension. In a national cohort study of African American and white adults in the United States aged ≥45 years and without prevalent hypertension, known as the REGARDS study (31), better cardiovascular health (quantified by higher levels of cumulative LS7 scores) was associated with a lower incidence of hypertension over a 9-year follow-up period. The incidence of hypertension was 75% for those with inadequate LS7, 47% for those with intermediate LS7, and 33% for those with optimal LS7. Akinyelure et al. (22) conducted a comprehensive analysis of the Jackson Heart Study and the REGARDS Study, suggesting a lower proportion of participants with aTRH and uncontrolled aTRH in those with more ideal LS7 scores. In the complete case analysis, which included participants taking class 1 or 2 antihypertensive medications at baseline with uncontrolled blood pressure and excluded participants with chronic kidney disease, those with more ideal LS7 scores had a lower proportion of participants with aTRH and uncontrolled aTRH.

The detailed analysis in this study examined the relationship between the components of LE8 and aTRH. Participants with moderate-to-ideal physical activity levels, BMI, and fasting blood glucose exhibited a significantly lower incidence of aTRH than those with poorer levels. However, tobacco/nicotine exposure, diet, sleep, and non-high-density lipoprotein cholesterol were not associated with aTRH events. Each component of LE8 represents cardiovascular health, with many components serving as modifiable risk factors for hypertension.

Relevant guidelines suggest further blood pressure reduction through nonpharmacological interventions such as lifestyle modifications; however, there is limited research assessing the effectiveness of nonpharmacological interventions in preventing hypertension. Compelling evidence indicates an inverse relationship between physical activity and the incidence of hypertension in adults with normal blood pressure and prehypertensive individuals (32). Additionally, emerging evidence suggests that physically active hypertensive patients can significantly improve their health-related quality of life and the progression of cardiovascular disease (33). A 12-week aerobic exercise program reduced the blood pressure of patients with resistant hypertension, thereby establishing moderate-intensity aerobic exercise as a standard adjunctive therapy for this patient population (34). The literature does not provide clear evidence of a direct causal relationship between smoking and hypertension. This concept is supported by the absence of evidence demonstrating a decrease in blood pressure values following long-term smoking cessation (35). However, hypertensive smokers are more prone to severe hypertension, including malignant hypertension and renovascular hypertension, potentially attributed to accelerated atherosclerosis. The adoption of the Dietary Approaches to Stop Hypertension (DASH) diet, rich in vegetables, fruits, whole grains, low-fat dairy, low saturated and total fat, and free from added sugars, has been recommended as a crucial lifestyle change for reducing the incidence and mortality risk of hypertension patients (36). The key aspect of the standard DASH diet is limiting daily sodium intake to <2000 mg/day, as recommended by the World Health Organization. Moderately reducing sodium intake and increasing potassium intake can significantly lower blood pressure and prevent premature deaths in millions of people (37). Large-scale open-label, cluster-randomized controlled trials have suggested that salt substitutes are associated with lower rates of stroke, major cardiovascular events, and all-cause mortality than ordinary salt in populations with a history of stroke or hypertension in individuals aged 60 years and above (38). Current research on hypertension and diet primarily focuses on preventing and reducing blood pressure levels in hypertensive patients, without considering the independent effects of the DASH diet apart from other lifestyle changes. Research on the relationship between specific diets and resistant hypertension remains limited and necessitates high-quality clinical evidence for support. Obesity is closely associated with the occurrence of hypertension and resistant hypertension, with hypertension management guidelines emphasizing weight reduction as a key lifestyle intervention (21, 39, 40). Relevant evidence suggests that weight loss is associated with reduced blood pressure in normotensive adults and hypertensive patients (41, 42). A meta-analysis of the impact of obesity on hypertension risk, which incorporated 66,598 participants, indicated that the increased risk of hypertension is negatively correlated with the population's potential BMI (43). A large cross-sectional study in Japan suggested that the increasing prevalence of obesity may contribute to the high incidence of hypertension in Japan, with a significant dose‒response relationship between BMI and the risk of hypertension (44). However, the impact of weight loss on the blood pressure of patients with resistant hypertension has yet to be extensively studied.

Although sleep is widely recognized as an important factor in cardiovascular health, our analysis revealed no significant association between the LE8 sleep component and aTRH. This finding suggests that the relationship between sleep and treatment-resistant hypertension may be more complex than previously understood, possibly influenced by factors such as assessment methods, variables and sleep quality. An experimentally induced sleep deprivation study (45) suggested that individuals with resistant hypertension have shorter total and rapid-eye-movement sleep time and lower sleep efficiency than subjects with the same severity and controlled hypertension or normal blood pressure. Independent of patients with sleep apnea, the experiment indicates that reduced sleep time can increase the severity of hypertension. However, current experimental results are based on basic experiments, and there is a lack of large-scale clinical studies to further describe sleep health, including habitual sleep duration, common sleep disorders (including insomnia), and disturbances in circadian rhythms, in relation to resistant hypertension. The treatment of aTRH is a comprehensive treatment plan that includes lifestyle improvements and medication control. Due to the lack of large-scale clinical research and the lack of correlation between LS8 and the risk of aTRH, our combined observations suggest that higher CVH levels are significantly associated with a reduced risk of aTRH. A higher LE8 score is the result of adherence to a comprehensive healthy lifestyle rather than a single healthy behavior. Future research needs to develop effective interventions to improve overall CVH and manage resistant hypertension.

The primary strengths of this study include the use of an updated LE8 score to assess CVH and a large study sample. However, our study has potential limitations. First, the assessment of LE8 behavioral factors relied on self-report questionnaires, introducing potential information bias such as measurement errors or misclassification. Second, although our blood pressure measurement data were derived from multiple blood pressure measurements within a short period, the long-term trends in blood pressure treatment and control necessitated repeated blood pressure measurements for accurately categorizing sustained hypertension status at the individual level, potentially introducing bias into our overall blood pressure estimations. Third, as a cross-sectional study, we were unable to determine causality in the association between LE8 and aTRH.

## Conclusion

In our cross-sectional study, we observed a significant association between higher LE8 scores and a lower risk of aTRH. Our findings suggest that implementing various healthy lifestyle practices and managing known cardiovascular risk factors could be a feasible comprehensive preventive approach to aTRH.

## Data Availability

The original contributions presented in the study are included in the article/Supplementary Material, further inquiries can be directed to the corresponding author/s.
