# Supplementary material for: Association between “Life's Essential 8” cardiovascular health and apparent treatment-resistant hypertension among US adults from the NHANES, 2005 to 2018
Source: Front Cardiovasc Med. 2024 Dec 13;11:1453563. doi: 10.3389/fcvm.2024.1453563 (PMC11671479; doi:10.3389/fcvm.2024.1453563)
Supplement: Supplementary file 1 [file Datasheet1.pdf]

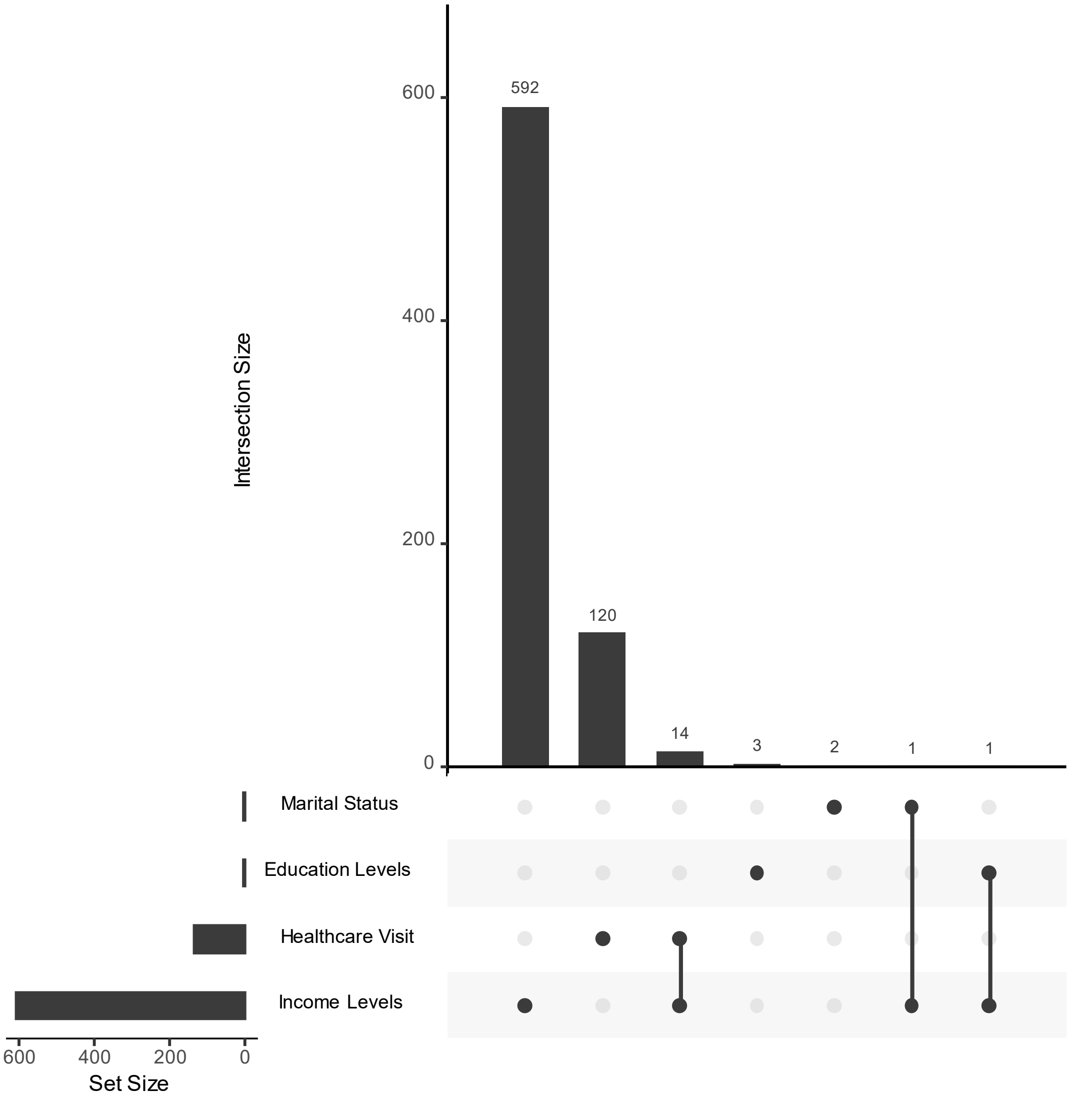

Figure S1 Patterns of missing data

Subgroups	n/N	OR[95% CI]	n/N	OR[95% CI]	$P_{Interaction}$
**Age, y**					0.075
<65	2554/3763	0.48[0.38,0.6]	161/3763	0.16[0.06,0.43]	
≥65	2708/3711	0.59[0.49,0.72]	202/3711	0.3[0.17,0.52]	
**Gender**					0.859
Female	2717/3935	0.51[0.42,0.63]	190/3935	0.24[0.12,0.49]	
Male	2545/3539	0.56[0.45,0.69]	173/3539	0.24[0.12,0.47]	
**Ethnicity**					0.911
Other	790/1128	0.56[0.37,0.86]	88/1128	0.39[0.14,1.05]	
Non-Hispanic White	2635/3596	0.49[0.39,0.62]	199/3596	0.25[0.13,0.46]	
Non-Hispanic Black	1294/1981	0.52[0.41,0.66]	58/1981	0.1[0.02,0.41]	
Mexican American	543/769	0.65[0.38,1.1]	18/769		
**Marital Status**					0.172
Not married nor living with a partner	2030/2996	0.57[0.46,0.71]	101/2996	0.34[0.16,0.73]	
Married or living with a partner	3232/4478	0.5[0.41,0.61]	262/4478	0.19[0.1,0.36]	
**Income Levels**					0.958
Low	1755/2265	0.59[0.43,0.83]	201/2265	0.22[0.11,0.47]	
Median	2143/3034	0.51[0.41,0.64]	116/3034	0.3[0.14,0.63]	
High	1364/2175	0.53[0.41,0.68]	46/2175	0.19[0.05,0.82]	
**Healthcare Visits Times**					0.666
<4/year	2112/2835	0.48[0.36,0.64]	176/2835	0.25[0.12,0.54]	
≥4/year	3150/4639	0.55[0.46,0.65]	187/4639	0.22[0.12,0.41]	
**Education Levels**					0.744
Less than high school	1223/1911	0.51[0.39,0.67]	44/1911	0.3[0.09,1.02]	
High school graduate or higher	4039/5563	0.54[0.45,0.64]	319/5563	0.23[0.13,0.39]	
**Chronic Kidney Diseases**					0.316
No	4034/5668	0.53[0.44,0.64]	310/5668	0.28[0.16,0.46]	
Yes	1228/1806	0.54[0.42,0.69]	53/1806	0.09[0.02,0.4]	

Figure S2 Odds Ratios for Middle and High Levels of Life's Essentials Compared to Low Level in Subgroup Analyses. $P_{Interaction}$, p values for interaction. For binary variables (age, gender, marital status, healthcare visits times, education levels, and chronic kidney diseases), p-values of the interaction obtained by directly adding it to the model; for multiple category variables (ethnicity and income levels), p values of the difference between the model with interaction and the model without interaction compared by likelihood ratio test.

Table S1 Classes of Antihypertension Drugs [1]

Class		Drug
Diuretics	Thiazide or thiazide-type diuretics	Chlorthalidone
		Hydrochlorothiazide
		Indapamide
		Metolazone
	Diuretics-loop	Bumetanide
		Furosemide
		Torsemide
	Diuretics-potassium sparing	Amiloride
		Triamterene
	Diuretics-aldosterone antagonists	Eplerenone
		Spironolactone
Angiotensin-converting enzyme inhibitors		Benazepril
		Captopril
		Enalapril
		Fosinopril
		Lisinopril
		Moexipril
		Perindopril
		Quinapril
		Ramipril
		Trandolapril
Angiotensin receptor blocker		Azilsartan
		Candesartan
		Eprosartan
		Irbesartan
		Losartan
		Olmesartan
		Telmisartan
		Valsartan
Calcium channel blockers (CCB)	CCB-dihydropyridines	Amlodipine
		Felodipine
		Isradipine
		Nicardipine SR
		Nifedipine LA
		Nisoldipine
	CCB-non dihydropyridines	Diltiazem ER
		Verapamil IR
		Verapamil SR
		Verapamil-delayed onset ER

Beta Blockers	Beta blockers-cardioselective	Atenolol
		Betaxolol
		Bisoprolol
		Metoprolol tartrate
		Metoprolol succinate
	Beta blockers- cardioselective and vasodilatory<br>Beta blockers-noncardioselective	Nebivolol
		Nadolol
		Propranolol IR
		Propranolol LA
	Beta blockers-intrinsic sympathomimetic activity	Acebutolol
		Penbutolol
		Pindolol
	Beta blockers-combined alpha- and beta-receptor	Carvedilol
		Carvedilol phosphate
		Labetalol
Direct renin inhibitor		Aliskiren
Alpha-1 blockers		Doxazosin
		Prazosin
		Terazosin
Central aloha2-agonist and other centrally acting drugs		Clonidine oral
		Clonidine patch
		Methyldopa
		Guanfacine
Direct Vasodilators		Hydralazine
		Minoxidil

IR, immediate release; LA, long-acting; and SR, sustained release

Table S2 Calculation method for life's essential 8 [2]

Life's Essential 8 metric	Method of measurement	Quantification of Life's Essential 8 metric
Diet	Measurement: healthy eating index-2015 (HEI-2015)	Quantiles of HEI-2015 (population)<br>Points Quantile<br>100 ≥95th percentile (top/ideal diet)<br>80 75th–94th percentile<br>50 50th–74th percentile<br>25 25th–49th percentile<br>0 1st–24th percentile (bottom/least ideal quartile)
Physical Activity (PA)	Measurement: Self-reported minutes of moderate or vigorous PA per week<br>Example tools for measurement: NHANES PAQ-K questionnaire[3]	Metric: Minutes of moderate- (or greater) intensity activity per week<br>Scoring:<br>Points Minutes<br>100 ≥150<br>90 120–149<br>80 90–119<br>60 60–89<br>40 30–59<br>20 1–29<br>0 0
Nicotine exposure	Measurement: Self-reported use of cigarettes or inhaled NDS<br>Example tools for measurement: NHANES SMQ[4]	Metric: Combustible tobacco use or inhaled NDS use; or secondhand smoke exposure<br>Scoring:<br>Points Status<br>100 Never smoker

		75 Former smoker, quit ≥5 y<br>50 Former smoker, quit 1–<5 y<br>25 Former smoker, quit <1 y, or currently using inhaled NDS<br>0 Current smoker<br>Subtract 20 points (unless score is 0) for living with active indoor smoker in home
Sleep health	Measurement: Self-reported average hours of sleep per night<br>Example tools for measurement: “On average, how many hours of sleep do you get per night?”<br>Consider objective sleep/ actigraphy data from wearable technology if available	Metric: Average hours of sleep per night<br>Scoring:<br>Points Level<br>100 7–<9<br>90 9–<10<br>70 6–<7<br>40 5–<6 or ≥10<br>20 4–<5<br>0 <4
Body Mass Index	Measurement: Body weight (kilograms) divided by height squared (meters squared) Example tools for measurement: Objective measurement of height and weight	Metric: BMI (kg/m2)<br>Scoring:<br>Points Level<br>100 <25<br>70 25.0–29.9<br>30 30.0–34.9<br>15 35.0–39.9<br>0 ≥40.0
Blood lipids	Measurement: Plasma total and HDL cholesterol with calculation of non–HDL	Metric: Non–HDL cholesterol (mg/dL)<br>Scoring:

	cholesterol Example tools for measurement: Fasting or non-fasting blood sample	Points Level<br>100 <130<br>60 130–159<br>40 160–189<br>20 190–219<br>0 ≥220<br>If drug-treated level, subtract 20 points
Blood glucose	Measurement: FBG or casual HbA1c<br>Example tools for measurement: Fasting (FBG, HbA1c) or non-fasting (HbA1c) blood sample	Metric: FBG (mg/dL) or HbA1c (%)<br>Scoring:<br>Points Level<br>100 No history of diabetes and FBG <100 (or HbA1c <5.7)<br>60 No diabetes and FBG 100–125 (or HbA1c 5.7–6.4) (prediabetes)<br>40 Diabetes with HbA1c <7.0<br>30 Diabetes with HbA1c 7.0–7.9<br>20 Diabetes with HbA1c 8.0–8.9<br>10 Diabetes with Hb A1c 9.0–9.9<br>0 Diabetes with HbA1c ≥10.0
Blood Pressure (BP)	Measurement: Appropriately measured systolic and diastolic BPs<br>Example tools for measurement: Appropriately sized BP cuff	Metric: Systolic and diastolic BPs (mmHg)<br>Scoring:<br>Points Level<br>100 <120/<80 (optimal)<br>75 120–129/<80 (elevated)<br>50 130–139 or 80–89 (stage 1hypertension)<br>25 140–159 or 90–99

		0	≥160 or ≥100
		Subtract 20 points if treated level	

HEI-2015 is designed to be scored from zero to 100, and than Identify the set of foods under consideration, determine the amount of each relevant dietary constituent, and derive the pertinent densities and score each HEI component using the relevant standards.[5]

To assess self-reported PA, participants were asked the frequency and duration of recreational physical activities over the past 30 days.

The assessment of smoking status was conducted through the administration of questionnaires that inquired about cigarette consumption over a span of 30 days, as well as the utilization of e-cigarettes or other forms of tobacco within a 5-day timeframe.

Sleep health was assessed with interviewer-administered questions about sleep habits and disorders.

BMI was calculated as the weight in kilograms divided by the square of the height in meters from standardized height and weight measurements.

Blood samples were obtained and sent to central laboratories for the determination of blood lipids, plasma glucose, and hemoglobin A1c. BP was measured manually after 5 minutes of seated rest in a quiet room and determination of the maximal inflation level; 3 measurement attempts were recorded, and we averaged the readings after excluding.

Overall CVH was calculated for each individual by summing the scores for each of the 8 metrics together and dividing the total by 8, to provide a LE8 score ranging from 0 to 100

Table S3 Association between life's essential 8 and 1~2 antihypertensive drugs uncontrolled hypertension and 3~4 antihypertensive drugs uncontrolled hypertensions

Life's Essential 8	Model1[a]	P Values	Model2[a]	P Values	Model3[a]	P Values
Uncontrolled Hypertension in 1~2 Antihypertensive Drugs[b]						
Low	Ref	Ref	Ref	Ref	Ref	Ref
Median	0.63[0.54,0.72]	<0.001	0.62[0.54,0.73]	<0.001	0.65[0.56,0.76]	<0.001
High	0.39[0.24,0.64]	<0.001	0.4[0.24,0.66]	<0.001	0.46[0.28,0.76]	0.003
P Values for Trend	<0.001		<0.001		<0.001	
Uncontrolled Hypertension in 3~4 Antihypertensive Drugs[c]						
Low	Ref	Ref	Ref	Ref	Ref	Ref
Median	0.61[0.41,0.92]	0.017	0.61[0.4,0.93]	0.022	0.59[0.39,0.9]	0.015
High	0.1[0.04,0.26]	<0.001	0.08[0.03,0.24]	<0.001	0.07[0.02,0.2]	<0.001
P Values for Trend	<0.001		<0.001		<0.001	

[a] odd ratios (OR) and 95% confidence intervals (CI) were calculated by logistic regressions. b Uncontrolled Hypertension in 1~2 Antihypertensive Drugs was defined as hypertension which was uncontrolled with 1~2 antihypertensive drugs or using 3 or more antihypertensive drugs; c Uncontrolled Hypertension in 3~4 Antihypertensive Drugs was defined as participants with resistant hypertension with uncontrolled hypertension in 4 antihypertensive drugs or using 5 or more antihypertensive drugs; Model1, unadjusted for covariates; Model2, adjusted for age, gender, ethnicity, marital status, income level, healthcare visit times last years, educational level and data cycles; Model3, Model2 +chronic kidney diseases.

Table S4 Association between life's essential 8 and resistant hypertension in sensitivity analyses

Life's Essential 8	Model1[a]	P Values	Model2[a]	P Values	Model3[a]	P Values
Cutoff= 140/90mmHg[b]						
Low	Ref	Ref	Ref	Ref	Ref	Ref
Median	0.49[0.42,0.57]	<0.001	0.49[0.41,0.57]	<0.001	0.5[0.43,0.59]	<0.001

High	0.25[0.15,0.4]	<0.001	0.26[0.16,0.43]	<0.001	0.28[0.17,0.47]	<0.001
P Values for Trend	<0.001		<0.001		<0.001	
Complete-case Analysis (n=6741)[c]						
Low	Ref	Ref	Ref	Ref	Ref	Ref
Median	0.49[0.42,0.57]	<0.001	0.49[0.42,0.58]	<0.001	0.51[0.44,0.6]	<0.001
High	0.2[0.12,0.34]	<0.001	0.22[0.13,0.36]	<0.001	0.24[0.14,0.39]	<0.001
P Values for Trend	<0.001		<0.001		<0.001	
Life’s Essential 8 in Continuous[d]						
Per 1 SD	0.64[0.6,0.69]	<0.001	0.63[0.58,0.68]	<0.001	0.64[0.59,0.7]	<0.001
Without Blood Pressure in LE8[e]						
Low	Ref	Ref	Ref	Ref	Ref	Ref
Median	0.6[0.51,0.71]	<0.001	0.6[0.51,0.72]	<0.001	0.63[0.53,0.74]	<0.001
High	0.45[0.34,0.6]	<0.001	0.46[0.34,0.62]	<0.001	0.5[0.36,0.67]	<0.001
P Values for Trend	<0.001		<0.001		<0.001	

[a] Odds ratios (OR) and their respective 95% confidence intervals (CI) were computed using logistic regression analysis. [b] The criteria for defining resistant hypertension were set at a blood pressure threshold of 140/90 mmHg. [c] Analyses were restricted to participants with complete data, excluding cases with missing data. [d] OR values were standardized for Life's Essential 8 by expressing the change in OR for every one standard deviation (SD). [e] Life's Essential 8 scores were calculated after excluding components related to blood pressure. Model1, unadjusted for covariates; Model2, adjusted for age, gender, ethnicity, marital status, income level, healthcare visit times last years, educational level and data cycles; Model3, Model2 +chronic kidney diseases.

References

1. Whelton, P.K., et al., *2017 ACC/AHA/AAPA/ABC/ACPM/AGS/APhA/ASH/ASPC/NMA/PCNA Guideline for the Prevention, Detection, Evaluation, and Management of High Blood Pressure in Adults: A Report of the American College of Cardiology/American Heart Association Task Force on Clinical Practice Guidelines.* J Am Coll Cardiol, 2018. **71**(19): p. e127-e248.
2. Lloyd-Jones, D.M., et al., *Life's Essential 8: Updating and Enhancing the American Heart Association's Construct of Cardiovascular Health: A Presidential Advisory From the American Heart Association.* Circulation, 2022. **146**(5): p. e18-e43.
3. Raghuveer, G., et al., *Cardiovascular Consequences of Childhood Secondhand Tobacco Smoke Exposure: Prevailing Evidence, Burden, and Racial and Socioeconomic Disparities: A Scientific Statement From the American Heart Association.* Circulation, 2016. **134**(16): p. e336-e359.
4. Iranpour, S. and S. Sabour, *Inverse association between caffeine intake and depressive symptoms in US adults: data from National Health and Nutrition Examination Survey (NHANES) 2005-2006.* Psychiatry Res, 2019. **271**: p. 732-739.
5. Krebs-Smith, S.M., et al., *Update of the Healthy Eating Index: HEI-2015.* J Acad Nutr Diet, 2018. **118**(9): p. 1591-1602.
